# Plasma MicroRNA Pair Panels as Novel Biomarkers for Detection of Early Stage Breast Cancer

**DOI:** 10.3389/fphys.2018.01879

**Published:** 2019-01-08

**Authors:** Rui Fang, Yong Zhu, Ling Hu, Vedbar Singh Khadka, Junmei Ai, Hanqing Zou, Dianwen Ju, Bin Jiang, Youping Deng, Xiamin Hu

**Affiliations:** ^1^Bioinformatics Core, Department of Complementary and Integrative Medicine, John A. Burns School of Medicine, University of Hawai‘i at Mānoa, Honolulu, HI, United States; ^2^Shanghai University of Medicine and Health Sciences, Shanghai, China; ^3^National Medical Centre of Colorectal Disease, The Third Affiliated Hospital of Nanjing University of Chinese Medicine, Nanjing, China; ^4^Department of Anesthesiology, Tianyou Hospital, Wuhan University of Science and Technology, Wuhan, China; ^5^Presence Health, Des Plaines, IL, United States; ^6^Department of Anatomy and Cell Biology, Rush University Medical Center, Chicago, IL, United States; ^7^Department of Microbiological and Biochemical Pharmacy, School of Pharmacy, Fudan University, Shanghai, China

**Keywords:** MicroRNA, plasma, biomarker, breast cancer, diagnosis

## Abstract

**Introduction:** Breast cancer is the second leading cause of cancer death among females. We sought to identify microRNA (miRNA) markers in breast cancer, and determine whether miRNA expression is predictive of early stage breast cancer. The paired panel of microRNAs is promising.

**Methods:** Global miRNA expression profiling was performed on three pooling samples of plasma from breast cancer, benign lesion and normal, using next generation sequencing technology. Thirteen microRNAs (hsa-miR-21-3p, hsa-miR-192-5p, hsa-miR-221-3p, hsa-miR-451a, hsa-miR-574-5p, hsa-miR-1273g-3p, hsa-miR-152, hsa-miR-22-3p, hsa-miR-222-3p, hsa-miR-30a-5p, hsa-miR-30e-5p, hsa-miR-324-3p, and hsa -miR-382-5p) were subsequently validated using real-time quantitative reverse transcription-polymerase chain reaction (RT-qPCR) in a cohort of 53 breast cancer, 40 benign lesions and 38 normal cases. The pairwise miRNA ratios were calculated as biomarkers to classify breast cancer.

**Results:** According to the model used to predict breast cancer from benign lesions, a panel of five miRNA pairs had high diagnostic power with an AUC of 0.942. The sensitivity, specificity, positive predictive value (PPV) and negative predictive value (NPV) of this model after 10-fold cross validation were 0.881, 0.775, 0.827, and 0.756, respectively. In addition, the other panels of miRNA pairs distinguishing the breast cancer from normal and non-cancer patients had good performance.

**Conclusion:** Certain MicroRNA pairs were identified and deemed effective in breast cancer screening, especially when distinguishing cancer from benign lesions.

## Introduction

According to the American Cancer Society report of 2017, an estimated 252,710 cases of invasive breast cancer and an additional 63,420 new cases of *in situ* lesions of the breast were diagnosed in women. It was further estimated that 40,610 women would die from breast cancer (American Cancer Society, 2017). Breast cancer is the second leading cause of cancer death in women following lung cancer. Sometimes breast cancer is found after the symptoms, but in many women breast cancer appear with no symptoms. Thus, the early diagnosis of breast cancer plays a critical role in the prognosis of breast cancer. Mammograms are currently the best test for breast cancer screening, however, the false positive rate is high. On average, 10% of women will be recalled from screening examinations for further testing such as the expensive magnetic resonance imaging (MRI) and/or the invasive biopsy, while only 5% of these women will actually have cancer (Rosenberg et al., 2006). According to one US study, over the course of 10 screening examinations, about 1.5 women will experience a false positive and about 19% will undergo biopsy (Elmore et al., 1998). It was estimated that breast cancer was over-diagnosed by mammography in up to 30% of all breast cancers diagnosed in 2008 (Jorgensen et al., 2009; Nelson et al., 2009; Puliti et al., 2009; Duffy et al., 2010; Bleyer and Welch, 2012; Gotzsche and Jorgensen, 2013; Marmot et al., 2013). Due to their low sensitivity, the known serum-based markers such as CA15.3 and BR27.29 are not used for screening breast cancer (Molina et al., 2005). Thus, there is a need for the development of novel biomarkers that are minimally invasive to improve the early diagnosis of malignant breast lesions.

MicroRNAs (miRNAs), a class of small non-coding RNAs (ncRNAs) containing ∼22 nucleotides, regulate gene expression in the post-transcription phase. They function in numerous cancer related processes such as cell proliferation, differentiation and apoptosis (Jansson and Lund, 2012). Clinical trials using circulating miRNAs as cancer biomarkers are being carried out in the United States and other countries ^1^. In recent years, with the advent of gene expression profiling technologies, an increasing number of studies have revealed the genetic association between miRNAs and cancer, including colorectal cancer (Schetter and Harris, 2009), lung cancer (Hu et al., 2016) and breast cancer (Takahashi et al., 2015; Kurozumi et al., 2017). However, very few studies have compared the expression profiles of miRNAs between benign lesions and breast cancer. Even though there were several reports using circulating miRNA markers for breast cancer detection, they were quite inconsistent (Roth et al., 2010; Cookson et al., 2012; Chan et al., 2013; Cuk et al., 2013; Guo et al., 2013; Freres et al., 2016).

Due to the low concentration of circulating ncRNAs in peripheral blood, data normalization in plasma or serum ncRNA experiments using real-time quantitative reverse transcription-polymerase chain reaction (RT-qPCR) is challenging. Current normalization strategy uses endogenous controls that display stable expression across all samples, like reference miRNAs such as miR-16 (Van Schooneveld et al., 2012) and miR-39 (Chen et al., 2016). Some researchers also have made effort to seek the suitable endogenous control miRNAs (ECMs) but no such suitable and universal ECMs have been established for blood miRNA quantification in humans (Davoren et al., 2008; Hackenberg et al., 2011; Langmead and Salzberg, 2012). Since there are no current consensus normalization methods for miRNAs, some studies have analyzed plasma miRNA values looking at the reciprocal ratio of miRNAs to bypass the normalization issue which has proven to be more informative for disease status than the absolute levels of individual miRNAs (Dou et al., 2018).

In this study, we proposed a ratio based method for breast cancer detection. We first performed the Illumina platform to sequence miRNAs in pooled samples and the selected miRNAs were further evaluated by RT-qPCR. The RT-qPCR was performed in a cohort of breast cancer, benign lesions, and normal patients and then calculated the pairwise ratio of any two miRNAs in the same samples. A diagnostic test based on the miRNA ratios was then constructed. This study focused on distinguishing cancer patients from benign lesions.

## Materials and Methods

### Patients, Plasma Sample Collection and Preparation

Each pooling sample contained 30 individual plasma samples from breast cancer, benign lesions, and normal patients, respectively, with matched age and race (Supplementary Table S1). The cohort included 53 breast cancer, 40 benign, and 38 normal patients from Rush Breast Cancer Repository (ORA number: 15021301-IRB01-CR02). The patients were selected according to the following criteria: (1) all patients were female; (2) all patients were diagnosed and confirmed by pathology; (3) patients with breast cancer were at the early stage (0, I, and II) according to the clinical staging method; (4) none of the patients underwent preoperative adjuvant chemotherapy or radiotherapy; and (5) patients had no other cancer or diseases which might affect the miRNA profiling. Benign lesions were defined as hyperplasia, fibroadenomas, cyst, and some unspecified findings in the breast. Normal blood samples were collected from healthy women with no history of malignant diseases and no inflammatory conditions. All plasma samples were collected using EDTA-anticoagulant tubes at 4,000 RPM for 10 min, followed by a 15 min high-speed centrifugation at 12,000 RPM to completely remove cell debris. The supernatant plasma was stored at –80°C until analysis.

### RNA Isolation and Illumina Next-Generation Sequencing

Total RNA was extracted from 200 μl of plasma using Qiagen miRNeasy Mini kit (Qiagen, Valencia, CA) according to the manufacturer’s protocol. In brief, the plasma was mixed with QIAzol Lysis Reagent and chloroform. After centrifugation, the aqueous phase was transferred into another tube, and 1.5 volumes of absolute ethanol were added. The mixture was then applied to miRNeasy Mini kit columns, followed by washing with RWT and RPE buffers. The RNAs were finally eluted in 40 μl of RNase-free water. Sequencing was performed on a HiSeq 2500 (Illumina). The sequencing adapters were removed from the FASTQ files by local alignment of the adapter to the sequenced reads. All sequences had a length <15 bp after the adapter removal was discarded. The reads in each library were summarized to tag in a quantified FASTA format. The FASTA reads were then mapped to the genome under consideration with Bowtie. To eliminate the ambiguous mapping hits, only the uniquely mapping loci with the newest alignment mismatches were reported, allowing for a maximum of two mismatches. The clean reads were then re-mapped back to human small ncRNA using Bowtie, the small ncRNA abundance (count) was determined, and the annotation for each mapped locus was derived from ncRNA database such as miRBase (Supplementary Table S2). The abundance (count) data was normalized by DESeq normalization. The top miRNAs that had fold change > = 5 in any comparison among pooling samples were selected for further PCR validation.

### MicroRNA Validation by RT-qPCR

MiRNAs were measured using Taqman miRNA assay kits (Applied Biosystems, United States) according to the manufacturer’s protocol. RNA concentration was measured using Agilent 2100 Bioanalyzer. Briefly, about 30 ng enriched RNA was reverse transcribed with a TaqMan microRNA Reverse Transcription Kit (Applied Biosystems, United States) in a 15 μL reaction volume. Expression levels of ncRNAs were quantified in triplicate by qRT-PCR using human TaqMan MicroRNA Assay kits (Applied Biosystems, Foster City, CA, United States) on an Eppendorf iplex 4 system (Eppendorf, Hamburg, Germany). The relative expression levels were express cycle threshold (CT) values. The ratio strategy described below in the statistical analysis section was used to reduce the experimental variations instead of normalizing by endogenous control.

### Statistical Analysis

CT values in PCR is a log (base 2) value of the observed count. From the formula below, we can see that the log (base 2) ratio value of two miRNAs is the difference in CT values of the two miRNAs, which will make the calculation even easier and more convenient for clinical practice based on RT-qPCR data.

$$\begin{array}{c} {\mathrm{Log}}_{2}(miRNA1/miRNA2){= Log}_{2}(2^{-CTmiRNA1}/2^{-CTmiRNA2})\\ {= Log}_{2}(2^{-CTmiRNA1+CTmiRNA2})\\ = CTmiRNA2 - CTmiRNA1 \end{array}$$

The difference in miRNA ratios between breast cancer and non-cancer patients (normal, benign or normal and benign patients) were examined by two sample *t*-tests. The fold change and regulation direction were then reported. The *p*-values were corrected by False Discover Rate (FDR) Benjamini and Hochberg. The association between the outcome variable, benign lesions or breast cancer, and each of the miRNA ratios were then evaluated by the logistic regression. The performance parameters such as sensitivity, specificity, positive predictive value (PPV) and negative predictive value (NPV) were summarized, and the area under the receiver operating (ROC) curve (AUC) was calculated to assess the discrimination power of each ratio. To avoid over-fitting, 10-fold cross validation was conducted. All analyses were performed by SAS 9.4 and *p*-value < 0.05 was considered as statistical significance. The miRNA pathway was analyzed using DIANA tools (Vlachos et al., 2015).

## Results

### Patients’ Characteristics

A total of 131 women which included 53 patients with early stage breast cancer, 40 patients with benign lesions and 38 normal patients were enrolled in the study The patients’ characteristics is summarized in Table 1. The mean age was 45.9 (*SD* = 10.5) years old in benign lesions group, which was significantly younger than the breast cancer (mean = 61.0, *SD* = 13.3) and normal (mean = 60.9, *SD* = 11.6) groups (*p* < 0.0001). There were 49 (92.5%) Caucasians in the cancer group, 37 (92.5%) in the benign lesions group and 36 (94.7%) in normal group (*p* = 1.00). The composition based on stages of breast cancer was as follows: 11 (20.8%) patients were stage 0, 36 (67.9%) were stage I, and 6 (11.3%) patients were stage II. Forty-one (41, 79.2%) cancer patients were invasive and 11 (20.8%) were *in situ*.

**Table 1:** The characteristics of the normals, patients with benign lesion and cancer.

	Breast cancer (*n* = 53)	Benign lesion (*n* = 40)	Normal (*n* = 38)	*p*-value
Age in year, mean (*SD*)	61.0 (13.3)	45.9 (10.5)	60.9 (11.6)	<0.0001
Race, *n* (%)				1.00
Caucasian	49	37	36	
Non-Caucasian	4	3	2	
Cancer stage				
0	11			
I	36			
II	6			
Cancer subtype				
Invasive	42			
*In situ*	11			

### MicroRNA Profiling of Plasma From Normal, Benign Lesions, and Breast Cancer Patients

By miRNA-sequencing, 190 miRNAs were identified from three pooled samples (one for each type of patient). The top miRNAs that had fold change ≥ 5 in any comparison were selected for further PCR validation. There were 13 miRNAs tested by RT-qPCR from a total of 131 plasma samples and they were hsa-miR-21-3p, hsa-miR-192-5p, hsa-miR-221-3p, hsa-miR-451a, hsa-miR-574-5p, hsa-miR-1273g-3p, hsa-miR-152, hsa-miR-22-3p, hsa-miR-222-3p, hsa-miR-30a-5p, hsa-miR-30e-5p, hsa-miR-324-3p, and hsa-miR-382-5p. Then pairwise miRNA ratios were calculated for each sample.

### Significantly Differentiated miRNA Pairs Among Normal, Benign Lesions, and Breast Cancer Patients

The concentrations of pairwise miRNA ratios were compared between breast cancer and three non-cancer groups (Cancer vs. Benign, Cancer vs. Normal, and Cancer vs. Non-cancer including Benign and Normal). MicroRNA ratios with fold change > 1.5 and FDR < 0.05 were listed in the Tables 2–4. The discriminative powers of individual ratios were ranged from 60 to 85%.

**Table 2:** The detection of miRNA ratios as potential biomarkers for diagnosis of early stage breast cancer (Cancer vs. Benign).

Ratio	Cancer	Benign	Fold Change	Regulation	*p*-value	FDR	AUC
**miRNA30a/miRNA382**	3.149	0.696	5.477	Up	2.1102E-05	0.0003	0.740
**miRNA192/miRNA382**	1.288	–1.073	5.138	Up	2.6838E-06	9.3932E-05	0.776
miRNA324/miRNA382	–0.984	–3.301	4.985	Up	2.3742E-06	9.3932E-05	0.784
miRNA21/miRNA382	4.570	2.296	4.836	Up	1.2653E-05	0.0003	0.736
miRNA1273g/miRNA382	1.712	–0.403	4.332	Up	2.1948E-05	0.0003	0.720
miRNA222/miRNA382	2.617	0.504	4.327	Up	5.7468E-05	0.0007	0.731
miRNA451/miRNA382	7.717	5.800	3.777	Up	0.0035	0.0192	0.641
miRNA152/miRNA382	–0.257	–2.139	3.684	Up	0.0001	0.0012	0.719
miRNA22/miRNA382	2.993	1.511	2.794	Up	0.006	0.0243	0.642
miRNA30e/miRNA382	1.616	0.144	2.773	Up	0.0038	0.0196	0.672
miRNA574/miRNA382	2.195	0.940	2.387	Up	0.0039	0.0196	0.606
**miRNA192/miRNA574**	–0.907	–2.013	2.152	Up	0.0034	0.0192	0.688
miRNA192/miRNA221	–1.299	–2.368	2.097	Up	0.0051	0.0233	0.658
miRNA21/miRNA574	2.375	1.357	2.026	Up	0.0051	0.0233	0.671
miRNA30a/miRNA30e	1.534	0.552	1.975	Up	2.3208E-06	9.3932E-05	0.801
**miRNA21/miRNA221**	1.983	1.001	1.974	Up	0.0003	0.0026	0.732
miRNA192/miRNA30e	–0.328	–1.217	1.853	Up	0.0113	0.0397	0.650
miRNA192/miRNA22	–1.705	–2.584	1.839	Up	0.0125	0.0422	0.648
miRNA21/miRNA30e	2.954	2.152	1.744	Up	0.0032	0.0192	0.684
miRNA21/miRNA22	1.577	0.786	1.731	Up	0.0011	0.0086	0.741
miRNA22/miRNA222	0.376	1.007	–1.549	Down	0.0083	0.0313	0.652
miRNA221/miRNA222	–0.030	0.791	–1.767	Down	0.0021	0.0139	0.698
miRNA574/miRNA1273g	0.483	1.343	–1.815	Down	0.0017	0.0117	0.703
miRNA22/miRNA30a	–0.156	0.815	–1.960	Down	1.7733E-05	0.0003	0.793
miRNA221/miRNA324	3.571	4.596	–2.035	Down	0.0055	0.0233	0.669
miRNA574/miRNA324	3.179	4.241	–2.088	Down	0.0114	0.0397	0.657
**miRNA221/miRNA30a**	–0.562	0.599	–2.236	Down	3.3603E-05	0.0004	0.763
miRNA574/miRNA30a	–0.955	0.244	–2.294	Down	0.0056	0.0233	0.686

*RT-qPCR tested miRNAs are in bold.*

**Table 3:** The detection of miRNA ratios as potential biomarkers for distinguishing breast cancer from normal patients.

Ratio	Cancer	Normal	Fold Change	Regulation	*p*-value	FDR	AUC
**miRNA324/miRNA382**	–0.984	–4.338	10.231	Up	1.2502E-08	1.3127E-06	0.845
miRNA30e/miRNA382	1.616	–0.959	5.957	Up	6.7281E-06	8.8307E-05	0.776
miRNA574/miRNA382	2.195	0.320	3.668	Up	0.0008	0.0043	0.699
miRNA152/miRNA382	–0.257	–2.110	3.612	Up	0.0024	0.0114	0.677
miRNA22/miRNA382	2.993	1.146	3.597	Up	0.0017	0.0083	0.664
miRNA30a/miRNA382	3.149	1.527	3.079	Up	0.006	0.0233	0.655
miRNA574/miRNA1273g	0.483	–0.499	1.975	Up	0.005	0.021	0.673
miRNA22/miRNA30e	1.378	2.105	–1.656	Down	0.0033	0.0151	0.666
miRNA21/miRNA22	1.577	2.406	–1.776	Down	0.0113	0.037	0.670
**miRNA30a/miRNA30e**	1.534	2.486	–1.935	Down	1.3784E-07	4.8246E-06	0.774
miRNA221/miRNA30e	0.972	2.115	–2.209	Down	0.0038	0.0168	0.687
miRNA222/miRNA30e	1.002	2.369	–2.580	Down	0.0001	0.0011	0.803
miRNA451/miRNA22	4.724	6.181	–2.747	Down	0.0079	0.0298	0.717
miRNA574/miRNA324	3.179	4.658	–2.789	Down	0.0107	0.0363	0.713
miRNA451/miRNA574	5.522	7.008	–2.801	Down	0.0087	0.0315	0.668
miRNA152/miRNA324	0.726	2.228	–2.832	Down	0.0007	0.0037	0.758
miRNA22/miRNA324	3.977	5.485	–2.844	Down	0.0002	0.0015	0.720
miRNA192/miRNA30e	–0.328	1.181	–2.845	Down	0.0003	0.002	0.770
miRNA21/miRNA30e	2.954	4.511	–2.942	Down	4.1527E-05	0.0004	0.768
miRNA1273g/miRNA30e	0.096	1.778	–3.208	Down	0.0005	0.003	0.739
miRNA30a/miRNA324	4.133	5.865	–3.322	Down	1.1736E-06	2.0538E-05	0.787
**miRNA221/miRNA324**	3.571	5.495	–3.793	Down	1.1750E-05	0.0001	0.753
miRNA222/miRNA324	3.601	5.748	–4.430	Down	2.4031E-07	6.3080E-06	0.812
miRNA451/miRNA30e	6.101	8.287	–4.549	Down	0.0003	0.002	0.746
miRNA192/miRNA324	2.272	4.560	–4.886	Down	6.2097E-07	1.3040E-05	0.809
**miRNA21/miRNA324**	5.554	7.891	–5.052	Down	1.0172E-07	4.8246E-06	0.810
miRNA1273g/miRNA324	2.695	5.157	–5.509	Down	6.4987E-06	8.8307E-05	0.816
miRNA451/miRNA324	8.700	11.666	–7.812	Down	9.9746E-06	0.0001	0.804

*RT-qPCR tested miRNAs are in bold.*

**Table 4:** The detection of miRNA ratios as potential biomarkers for distinguishing breast cancer from non-cancer (Normal +Benign) patients.

Ratio	Cancer	Normal+Benign	Fold Change	Regulation	*p*-value	FDR	AUC
miRNA324/miRNA382	–0.984	–3.807	7.076	Up	6.5987E-09	6.9287E-07	0.814
**miRNA30a/miRNA382**	3.149	1.101	4.137	Up	0.0001	0.0026	0.699
**miRNA30e/miRNA382**	1.616	–0.393	4.025	Up	4.6119E-05	0.0012	0.722
**miRNA152/miRNA382**	–0.257	–2.125	3.649	Up	0.0001	0.0026	0.698
**miRNA192/miRNA382**	1.288	–0.442	3.318	Up	0.0002	0.0033	0.692
miRNA222/miRNA382	2.617	0.945	3.187	Up	0.001	0.0079	0.675
miRNA21/miRNA382	4.570	2.908	3.164	Up	0.001	0.0079	0.664
miRNA22/miRNA382	2.993	1.333	3.160	Up	0.0018	0.0106	0.653
miRNA574/miRNA382	2.195	0.638	2.943	Up	0.0006	0.0061	0.651
miRNA1273g/miRNA382	1.712	0.192	2.867	Up	0.0018	0.0106	0.649
miRNA221/miRNA30a	–0.562	0.127	–1.612	Down	0.0078	0.0373	0.634
miRNA30e/miRNA324	2.599	3.413	–1.758	Down	0.0092	0.0419	0.620
miRNA152/miRNA324	0.726	1.682	–1.939	Down	0.0028	0.0139	0.660
miRNA192/miRNA324	2.272	3.364	–2.133	Down	0.0021	0.011	0.655
miRNA222/miRNA324	3.601	4.752	–2.221	Down	0.0006	0.0061	0.662
miRNA21/miRNA324	5.554	6.715	–2.236	Down	0.0005	0.0056	0.655
miRNA22/miRNA324	3.977	5.140	–2.239	Down	0.0012	0.0079	0.669
miRNA574/miRNA324	3.179	4.444	–2.404	Down	0.0021	0.011	0.684
miRNA1273g/miRNA324	2.695	3.999	–2.468	Down	0.0012	0.0079	0.677
**miRNA221/miRNA324**	3.571	5.034	–2.756	Down	1.3465E-05	0.0005	0.710
miRNA451/miRNA324	8.700	10.351	–3.139	Down	0.0012	0.0079	0.670

*RT-qPCR tested miRNAs are in bold.*

### Identification of miRNA Ratios as Biomarkers for the Detection of Breast Cancer

**FIGURE 1:**
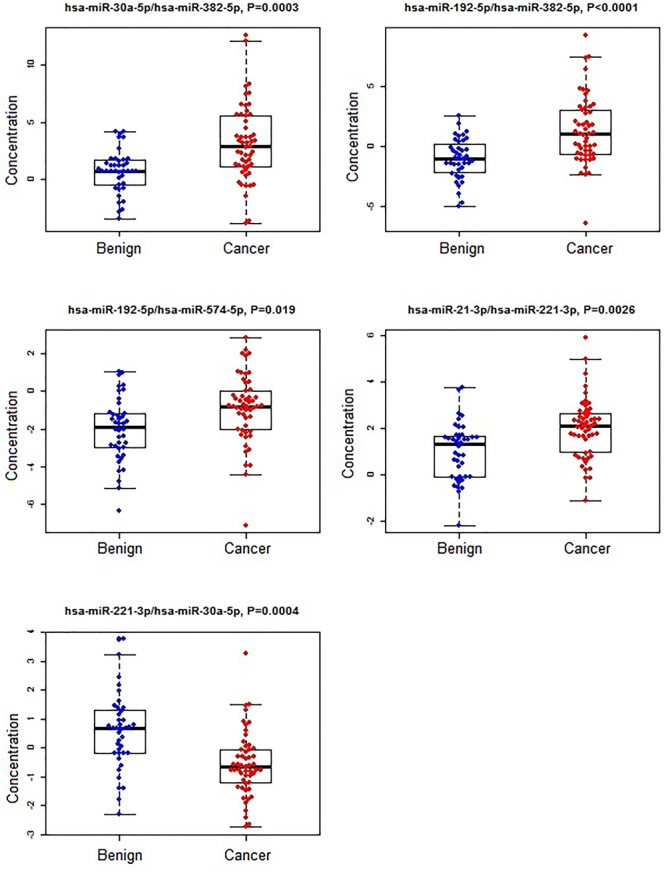
The concentrations of selected miRNA ratios between breast cancer and benign patients. The black horizontal bars are median values and the bottom and top box are the 25th and 75th percentiles. The *p*-values were reported by FDR.

**FIGURE 2:**
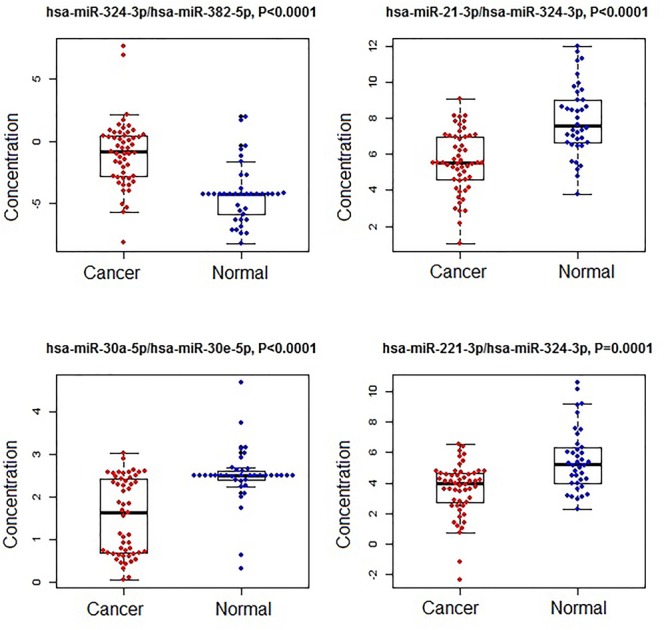
The concentrations of selected miRNA ratios between breast cancer and normal patients. The black horizontal bars are median values and the bottom and top box are the 25th and 75th percentiles. The *p*-values were reported by FDR.

**FIGURE 3:**
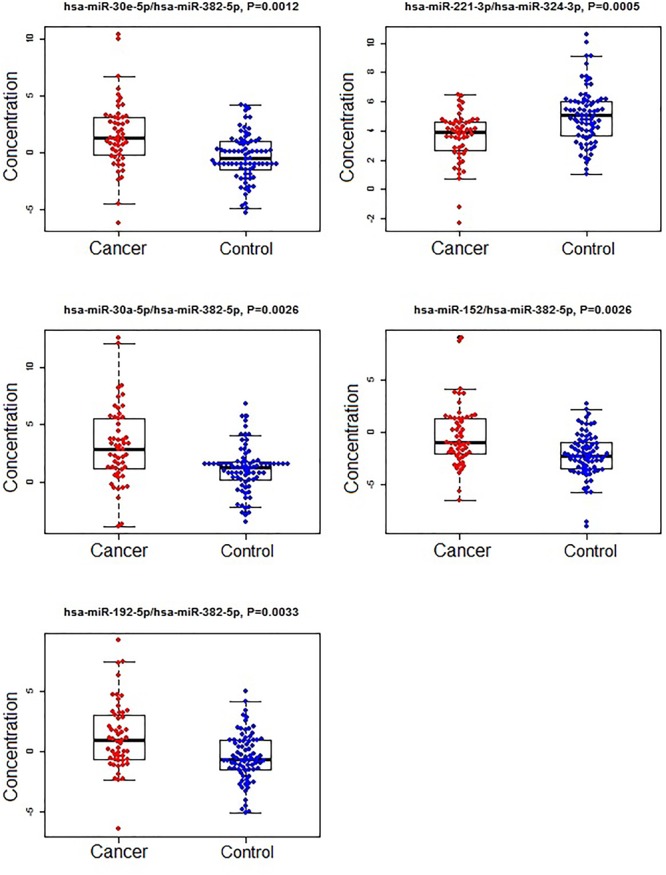
The concentrations of selected miRNA ratios between breast cancer and non-cancer (control, including benign and normal) patients. The black horizontal bars are median values and the bottom and top box are the 25th and 75th percentiles. The *p*-values were reported by FDR.

**FIGURE 4:**
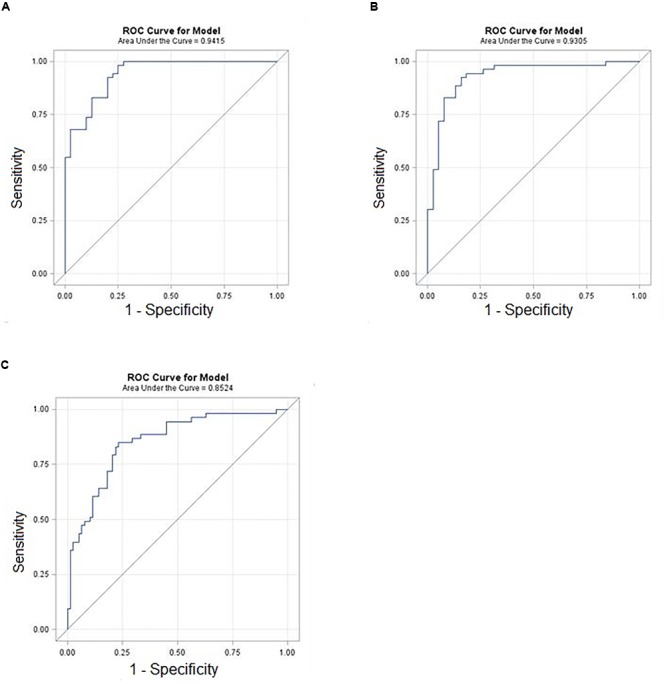
ROC curves of selected miRNA ratios with demographic (age) in the prediction of breast cancer. **(A)** Cancer vs. Benign. **(B)** Cancer vs. Normal. **(C)** Cancer vs. Control (Benign+Normal).

**FIGURE 5:**
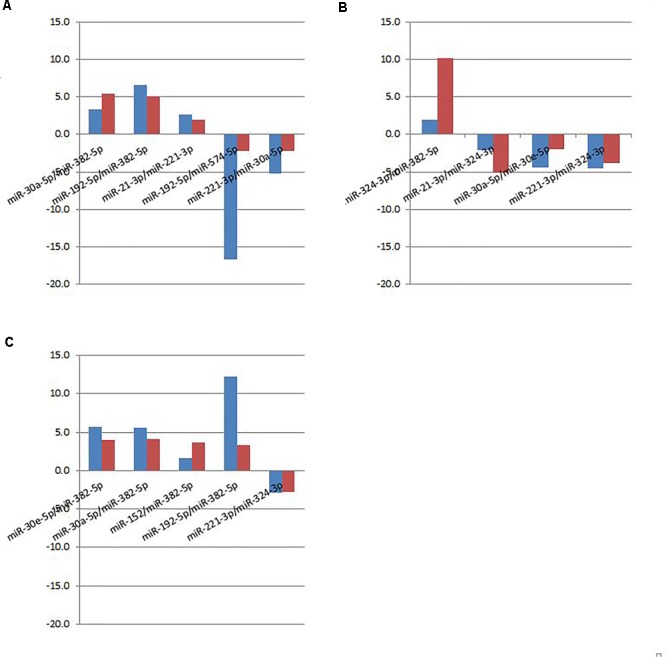
Fold changes of selected miRNA ratios for individual qPCR (red bar) and pooled sequencing (blue bar). **(A)** Cancer vs. Benign. **(B)** Cancer vs. Normal. **(C)** Cancer vs. Control (Benign+Normal).

A subset of ratios was chosen based on the rank of the correlation with the classification (cancer or not). Table 5 summarized the qPCR evaluation results in each comparison. The best miRNA ratio combination in different groups was listed in Table 5, and Figures 1–3 showed the expression values of each representative miRNA ratio markers in different groups. In particular, the best combination that distinguished the breast cancer from benign lesions was hsa-miR-30a-5p/hsa-miR-382-5p, hsa-miR-192-5p/hsa-miR-382-5p, hsa-miR-192-5p/hsa-miR-574-5p, hsa-miR-21-3p/hsa-miR-221-3p, and hsa-miR-221-3p/hsa-miR-30a-5p. This model with age as a confounder yielded an AUC of 0.942 (95% CI: 0.898 to 0.985, Figure 4A). After 10-fold cross validation, the sensitivity, specificity, PPV, NPV, and AUC were 0.881, 0.775, 0.827, 0.756, and 0.901, respectively. Similarly, the selected panel of miRNA ratios combined with age yielded AUCs of 0.931 (95% CI: 0.874 to 0.987) and 0.852 (95% CI: 0.786 to 0.919) under comparison between breast cancer and normal/non-cancer groups (Figures 4B,C). The sensitivity, specificity, PPV, NPV, and AUC after 10-fold cross-validation were presented in Table 2. Interestingly, these selected miRNA ratios from individual qPCR had consisted fold changes with the sequence results from pooling samples (Figures 5A–C).

**Table 5:** PCR evaluation of paired miRNA ratios in individual samples.

	Cancer vs. Benign^∗^ (53 vs. 40)	Cancer vs. Normal^∗^ (53 vs. 38)	Cancer vs. Control (Benign + Normal) (53 vs. 78)
Number of ratios that significantly identified with fold change > 1.5	28 (Table 2)	28 (Table 3)	21 (Table 4)
Number of ratios selected in the final model	5	4	5
Name of ratios selected in the final model	hsa-miR-30a-5p/hsa-miR-382-5p, hsa-miR-192-5p/hsa-miR-382-5p, hsa-miR-192-5p/hsa-miR-574-5p, hsa-miR-21-3p/hsa-miR-221-3p, hsa-miR-221-3p/miR-30a-5p (Figure 1)	hsa-miR-324-3p/hsa-miR-382-5p, hsa-miR-21-3p/hsa-miR-324-3p, hsa-miR-30a-5p/has-miR-30e-5p, hsa-miR-221-3p/has-miR-324-3p (Figure 2)	hsa-miR-30e-5p/hsa-miR-382-5p, hsa-miR-221-3p/hsa-miR-324-3p, hsa-miR-30a-5p/hsa-miR-382-5p, hsa-miR-152/hsa-miR-382-5p, hsa-miR-192-5p/hsa-miR-382-5p (Figure 3)
Sensitivity	0.881	0.890	0.717
Specificity	0.775	0.925	0.782
PPV	0.827	0.889	0.691
NPV	0.756	0.891	0.803
AUC	0.901	0.901	0.820

*^∗^Age as a co-founder was included in the model since it significantly differed between two groups. Sensitivity, specificity, PPV, NPV, and AUC were obtained from the final model after 10-fold cross-validation.*

### MicroRNA Function Analysis

Based on the model that predicted the breast cancer from benign lesions, six miRNAs involved were hsa-miR-30a-5p, hsa-miR-382-5p, hsa-miR-192-5p, hsa-miR-574-5p, hsa-miR-21-3p, and hsa-miR-221-3p. These miRNAs were found in cancer related pathways (Supplementary Figure S1) and targeted in thousands of overlapping genes. The partial pathways and targeted genes are presented in Supplementary Table S3.

## Discussion

In clinical practice, mammographic screening is the most common method used for early stage breast cancer diagnosis. However, high false positives rates in mammography warrants further investigation using expensive breast imaging and invasive biopsy exposing women to harmful anti-cancer therapy and affecting their quality of life. Therefore, the development of a more sensitive approach for early breast cancer diagnosis, particularly from benign lesions, is needed to supplement and/or complement existing detection methods. Our goal was to determine pairs of miRNA as alternative biomarkers that can be used to differentiate breast cancer from benign lesions or non-cancer patients. As far as we know, this is the first study on miRNA ratios in distinguishing early stage breast cancer from benign lesions. Our current data showed that the miRNA ratios are likely to perform well in distinguishing breast cancer from benign lesions. The miRNA ratio chosen over the single miRNA provided more candidates for diagnosis of early stage breast cancer. In addition, the combination of the selected miRNA ratios had a high diagnostic value for breast cancer prediction. We have identified five miRNA ratios that can differentiate breast cancer from benign lesions with over 90% accuracy. The ratio based normalization method, which is completely independent of spike-in or internal controls, has a great chance of producing more reliable and reproducible biomarkers in common types of cancer. In addition, the ratio based normalization method provides more biomarkers as candidates.

The interpretation of the miRNA ratios is more complicated than the individual miRNAs. Based on the equation in the methods section and Figures 1–3, the up-regulation of the miRNA ratio in the cancer group indicates higher level of the miRNA as the denominator in the ratio and lower level of the miRNA as the nominator in the ratio, and vice versa. For example, miR-192 was identified as the nominator in two ratios that distinguished breast cancer from benign lesions. This indicated that the concentration level was lower in cancer group, i.e., down regulation. The individual miRNAs from the ratios studied in our study were primarily identified in other studies. The miR-192 was found in down-regulation in breast cancer compared with the non-cancerous tissue, indicating that miR-192 may act as tumor suppressor gene in the development of breast cancer (Hu et al., 2013). Yang et al. (2017) reviewed the versatile functions of miR-30 family members in breast cancer. In particular, miR-30a suppressed tumor growth, proliferation, migration and invasion of breast cancer. Another study found that miR-221 was over expressed in breast cancer tissue compared to the non-cancerous tissue and concluded that miR-221 was a potential biomarker for predicting the survival of breast cancer patients (Eissa et al., 2015). However, plasma miR-21 expression was not observed to have a significant difference in benign patients, under normal controls, compared to cancer patients in breast cancer (Chen et al., 2016). Interestingly, our study shows that miR-21 interacted with other miRNAs can regulate significantly, indicating that miR-21 could serve as a long-term follow-up biomarker in the detection of cancer. Ho et al. study found that miR-382-5p was up-regulated in breast cancer compared to the benign breast disease, and significantly functioned as an independent oncomiR for the higher incidence and poorer prognosis of breast cancer (Ho et al., 2017). MiR-574-3p was first reported by Krishnan et al. as a promising prognostic maker for breast cancer (Krishnan et al., 2015). So far, nobody has released the relationship between miR-574-5p and breast cancer. Our study is a good start for further research.

We realized that the sample size of our subjects, including breast cancer patients, benign lesions, and normal controls are small, limiting the evaluation on miRNAs as predictive biomarkers in the early detection of cancer. Another limitation of this study is the lack of a validation patient cohort. We believe that further studies investigating more powerful and specific miRNA biomarkers to discriminate early cancer from pre-cancerous lesions are needed.

## Conclusion

The expression profile of plasma miRNA ratios can serve as novel non-invasive biomarkers for the early detection of breast cancer. The strategy of using next generation sequencing followed by RT-qPCR validation provides a successful approach to identifying plasma miRNA profiles as biomarkers for the diagnosis of common types of cancer.

## Availability of Data and Material

The datasets used and/or analyzed during the current study are available from the corresponding author upon reasonable request.

## Ethics Statement

The study was approved by the institutional review board of the Rush University Medical Center (ORANo. 15021301-IRB01-CR02). All participants provided a written informed consent.

## Author Contributions

RF, YD, YZ, LH, and JA analyzed the data. RF, VSK, and YD interpreted the results and drafted the manuscript. LH, DJ, and BJ helped in manuscript revision. YD, YZ, LH, HZ, and XH designed the study and experiments. All authors have read and approved the final manuscript.

## Conflict of Interest Statement

The authors declare that the research was conducted in the absence of any commercial or financial relationships that could be construed as a potential conflict of interest.

## Footnotes

1 clinicaltrials.gov

## Supplementary Material

FIGURE S1 Function analysis heat map showed the hierarchical clustering of miRNAs and pathways based on the levels of their interactions.

TABLE S1 The characteristics of the pooling samples.

TABLE S2 Raw count data derived from the Illumina next-generation sequencing experiment.

TABLE S3 Functional analysis/Ingenuity pathway analysis (DIANA tool).

## References

[B1] American Cancer Society (2017). *Cancer Facts and Figures 2017.* Atlanta, GA: American Cancer Society.

[B2] Bleyer A. Welch H. G. (2012). Effect of three decades of screening mammography on breast-cancer incidence. *N. Engl. J. Med.* 367 1998–2005. 10.1056/NEJMoa1206809 23171096

[B3] Chan M. Liaw C. S. Ji S. M. Tan H. H. Wong C. Y. Thike A. A. (2013). Identification of circulating microRNA signatures for breast cancer detection. *Clin. Cancer Res.* 19 4477–4487. 10.1158/1078-0432.CCR-12-3401 23797906

[B4] Chen H. Liu H. Zou H. Chen R. Dou Y. Sheng S. (2016). Evaluation of plasma miR-21 and miR-152 as diagnostic biomarkers for common types of human cancers. *J. Cancer* 7 490–499. 10.7150/jca.12351 26958084 PMC4780124

[B5] Cookson V. J. Bentley M. A. Hogan B. V. Horgan K. Hayward B. E. Hazelwood L. D. (2012). Circulating microRNA profiles reflect the presence of breast tumours but not the profiles of microRNAs within the tumours. *Cell Oncol.* 35 301–308. 10.1007/s13402-012-0089-1 22821209 PMC12995027

[B6] Cuk K. Zucknick M. Heil J. Madhavan D. Schott S. Turchinovich A. (2013). Circulating microRNAs in plasma as early detection markers for breast cancer. *Int. J. Cancer* 132 1602–1612. 10.1002/ijc.27799 22927033

[B7] Davoren P. A. Mcneill R. E. Lowery A. J. Kerin M. J. Miller N. (2008). Identification of suitable endogenous control genes for microRNA gene expression analysis in human breast cancer. *BMC Mol. Biol.* 9:76. 10.1186/1471-2199-9-76 18718003 PMC2533012

[B8] Dou Y. Zhu Y. Ai J. Chen H. Liu H. Borgia J. A. (2018). Plasma small ncRNA pair panels as novel biomarkers for early-stage lung adenocarcinoma screening. *BMC Genomics* 19:545. 10.1186/s12864-018-4862-z 30029594 PMC6053820

[B9] Duffy S. W. Tabar L. Olsen A. H. Vitak B. Allgood P. C. Chen T. H. (2010). Absolute numbers of lives saved and overdiagnosis in breast cancer screening, from a randomized trial and from the breast screening programme in England. *J. Med. Screen* 17 25–30. 10.1258/jms.2009.009094 20356942 PMC3104821

[B10] Eissa S. Matboli M. Sharawy A. El-Sharkawi F. (2015). Prognostic and biological significance of microRNA-221 in breast cancer. *Gene* 574 163–167. 10.1016/j.gene.2015.08.004 26253160

[B11] Elmore J. G. Barton M. B. Moceri V. M. Polk S. Arena P. J. Fletcher S. W. (1998). Ten-year risk of false positive screening mammograms and clinical breast examinations. *N. Engl. J. Med.* 338 1089–1096. 10.1056/NEJM199804163381601 9545356

[B12] Freres P. Wenric S. Boukerroucha M. Fasquelle C. Thiry J. Bovy N. (2016). Circulating microRNA-based screening tool for breast cancer. *Oncotarget* 7 5416–5428. 10.18632/oncotarget.6786 26734993 PMC4868695

[B13] Gotzsche P. C. Jorgensen K. J. (2013). Screening for breast cancer with mammography. *Cochrane Database Syst. Rev.* CD001877. 10.1002/14651858.CD001877.pub5 23737396 PMC6464778

[B14] Guo L. Zhao Y. Yang S. Cai M. Wu Q. Chen F. (2013). Genome-wide screen for aberrantly expressed miRNAs reveals miRNA profile signature in breast cancer. *Mol. Biol. Rep.* 40 2175–2186. 10.1007/s11033-012-2277-5 23196705

[B15] Hackenberg M. Rodriguez-Ezpeleta N. Aransay A. M. (2011). miRanalyzer: an update on the detection and analysis of microRNAs in high-throughput sequencing experiments. *Nucleic Acids Res.* 39 W132–W138. 10.1093/nar/gkr247 21515631 PMC3125730

[B16] Ho J. Y. Hsu R. J. Liu J. M. Chen S. C. Liao G. S. Gao H. W. (2017). MicroRNA-382-5p aggravates breast cancer progression by regulating the RERG/Ras/ERK signaling axis. *Oncotarget* 8 22443–22459. 10.18632/oncotarget.12338 27705918 PMC5410235

[B17] Hu F. Meng X. Tong Q. Liang L. Xiang R. Zhu T. (2013). BMP-6 inhibits cell proliferation by targeting microRNA-192 in breast cancer. *Biochim. Biophys. Acta* 1832 2379–2390. 10.1016/j.bbadis.2013.08.011 24012720

[B18] Hu L. Ai J. Long H. Liu W. Wang X. Zuo Y. (2016). Integrative microRNA and gene profiling data analysis reveals novel biomarkers and mechanisms for lung cancer. *Oncotarget* 7 8441–8454. 10.18632/oncotarget.7264 26870998 PMC4890978

[B19] Jansson M. D. Lund A. H. (2012). MicroRNA and cancer. *Mol. Oncol.* 6 590–610. 10.1016/j.molonc.2012.09.006 23102669 PMC5528350

[B20] Jorgensen K. J. Zahl P. H. Gotzsche P. C. (2009). Overdiagnosis in organised mammography screening in Denmark. A comparative study. *BMC Womens Health* 9:36. 10.1186/1472-6874-9-36 20028513 PMC2807851

[B21] Krishnan P. Ghosh S. Wang B. Li D. Narasimhan A. Berendt R. (2015). Next generation sequencing profiling identifies miR-574-3p and miR-660-5p as potential novel prognostic markers for breast cancer. *BMC Genomics* 16:735. 10.1186/s12864-015-1899-0 26416693 PMC4587870

[B22] Kurozumi S. Yamaguchi Y. Kurosumi M. Ohira M. Matsumoto H. Horiguchi J. (2017). Recent trends in microRNA research into breast cancer with particular focus on the associations between microRNAs and intrinsic subtypes. *J. Hum. Genet.* 62 15–24. 10.1038/jhg.2016.89 27439682

[B23] Langmead B. Salzberg S. L. (2012). Fast gapped-read alignment with Bowtie 2. *Nat. Methods* 9 357–359. 10.1038/nmeth.1923 22388286 PMC3322381

[B24] Marmot M. G. Altman D. G. Cameron D. A. Dewar J. A. Thompson S. G. Wilcox M. (2013). The benefits and harms of breast cancer screening: an independent review. *Br. J. Cancer* 108 2205–2240. 10.1038/bjc.2013.177 23744281 PMC3693450

[B25] Molina R. Barak V. Van Dalen A. Duffy M. J. Einarsson R. Gion M. (2005). Tumor markers in breast cancer- European group on tumor markers recommendations. *Tumour Biol.* 26 281–293. 10.1159/000089260 16254457

[B26] Nelson H. D. Tyne K. Naik A. Bougatsos C. Chan B. K. Humphrey L. (2009). Screening for breast cancer: an update for the U.S. preventive services task force. *Ann. Intern. Med.* 151 727–737. 10.7326/0003-4819-151-10-200911170-00009 19920273 PMC2972726

[B27] Puliti D. Zappa M. Miccinesi G. Falini P. Crocetti E. Paci E. (2009). An estimate of overdiagnosis 15 years after the start of mammographic screening in Florence. *Eur. J. Cancer* 45 3166–3171. 10.1016/j.ejca.2009.06.014 19879130

[B28] Rosenberg R. D. Yankaskas B. C. Abraham L. A. Sickles E. A. Lehman C. D. Geller B. M. (2006). Performance benchmarks for screening mammography. *Radiology* 241 55–66. 10.1148/radiol.2411051504 16990671

[B29] Roth C. Rack B. Muller V. Janni W. Pantel K. Schwarzenbach H. (2010). Circulating microRNAs as blood-based markers for patients with primary and metastatic breast cancer. *Breast Cancer Res.* 12:R90. 10.1186/bcr2766 21047409 PMC3046429

[B30] Schetter A. J. Harris C. C. (2009). Plasma microRNAs: a potential biomarker for colorectal cancer? *Gut* 58 1318–1319. 10.1136/gut.2009.176875 19749133

[B31] Takahashi R. U. Miyazaki H. Ochiya T. (2015). The roles of MicroRNAs in breast cancer. *Cancers* 7 598–616. 10.3390/cancers7020598 25860815 PMC4491673

[B32] Van Schooneveld E. Wouters M. C. Van Der Auwera I. Peeters D. J. Wildiers H. Van Dam P. A. (2012). Expression profiling of cancerous and normal breast tissues identifies microRNAs that are differentially expressed in serum from patients with (metastatic) breast cancer and healthy volunteers. *Breast Cancer Res.* 14:R34. 10.1186/bcr3127 22353773 PMC3496152

[B33] Vlachos I. S. Zagganas K. Paraskevopoulou M. D. Georgakilas G. Karagkouni D. Vergoulis T. (2015). DIANA-miRPath v3.0: deciphering microRNA function with experimental support. *Nucleic Acids Res.* 43 W460–W466. 10.1093/nar/gkv403 25977294 PMC4489228

[B34] Yang S. J. Yang S. Y. Wang D. D. Chen X. Shen H. Y. Zhang X. H. (2017). The miR-30 family: versatile players in breast cancer. *Tumour Biol.* 39:1010428317692204. 10.1177/1010428317692204 28347244

