# Supplementary figures and images for: Plasma MicroRNA Pair Panels as Novel Biomarkers for Detection of Early Stage Breast Cancer
Source: Front Physiol. 2019 Jan 8;9:1879. doi: 10.3389/fphys.2018.01879 (PMC6331533; doi:10.3389/fphys.2018.01879)

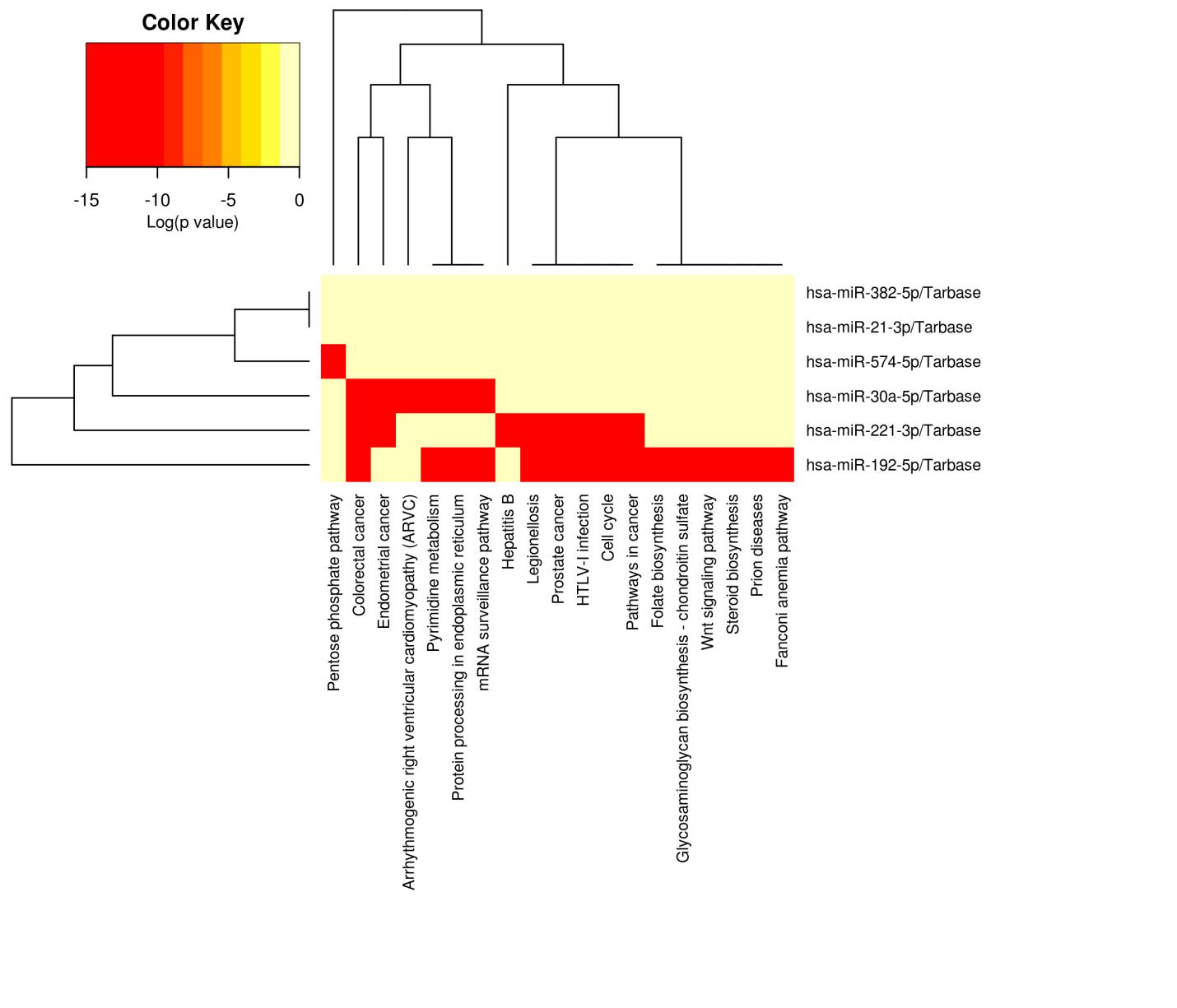

Supplement: FIGURE S1 — Function analysis heat map showed the hierarchical clustering of miRNAs and pathways based on the levels of their interactions. [file Image_1.jpeg]
