# Supplementary material for: Plasma MicroRNA Pair Panels as Novel Biomarkers for Detection of Early Stage Breast Cancer
Source: Front Physiol. 2019 Jan 8;9:1879. doi: 10.3389/fphys.2018.01879 (PMC6331533; doi:10.3389/fphys.2018.01879)
Supplement: TABLE S1 — The characteristics of the pooling samples. [file Table_1.docx]

Supplementary Table 1The characteristics of the pooling samples

|  | Breast cancer  (n=30) | Benign lesion  (n=30) | Normal  (n=30) |
| --- | --- | --- | --- |
| Age in year, mean (SD) | 58.5 (12.4) | 59.4.9 (11.65) | 61.1 (12.8) |
| Race, n (%) |  |  |  |
| Caucasian | 28 | 27 | 26 |
| Non-Caucasian | 2 | 3 | 4 |
| Cancer stage |  |  |  |
| 0 | 5 |  |  |
| I | 22 |  |  |
| II | 3 |  |  |
| Cancer subtype |  |  |  |
| Invasive | 25 |  |  |
| In situ | 5 |  |  |
