# Supplementary material for: Plasma MicroRNA Pair Panels as Novel Biomarkers for Detection of Early Stage Breast Cancer
Source: Front Physiol. 2019 Jan 8;9:1879. doi: 10.3389/fphys.2018.01879 (PMC6331533; doi:10.3389/fphys.2018.01879)
Supplement: TABLE S3 — Functional analysis/Ingenuity pathway analysis (DIANA tool). [file Table_3.docx]

Supplementary Table 3 Functional analysis/Ingenuity pathway analysis (DIANA tool)

| KEGG pathway | miRNA | Target genes | P-value |
| --- | --- | --- | --- |
| Cell cycle | hsa-miR-192-5p | RBL2, CDC14A, MCM6, ORC1, CDKN1B, CDKN2A, ANAPC10, E2F5, TTK, RB1, CDC7, CDC20, BUB1B, MAD2L1, CCNE1, CDKN2D, RAD21, MCM3, CDC25A | 5.12E-09 |
|  | hsa-miR-221-3p | CDKN1B, WEE1, CDKN1C, E2F3 |  |
|  | hsa-miR-30a-5p | YWHAE, YWHAZ, CDC20 |  |
| Pathway in cancer | hsa-miR-192-5p | FZD7, FOS, APC, CRK, RAD51, BCL2, CDKN1B, WNT3, BRCA2, IGF1R, CDKN2A, APPL1, RET, ITGAV, FZD4, MAPK9, MSH6, RB1, HSP90B1, FGF2, FZD1, PRARG, NKX3-1, CCNE1, TCF7, TFG | 1.10E-06 |
|  | hsa-miR-221-3p | FOS, CDKN1B, CSP3, CTNNB1, E2F3, KIT, PTEN, DVL2 |  |
|  | hsa-miR-30a-5p | WNT5A, BAX, IGF1R, EGFR, MLH1, JUN, CTNNB1, ITGA2, BCL2L1, ITGA6, MAPK1, JUP |  |
| Colorectal cancer | hsa-miR-192-5p | FOS, APC, BCL2, APPL1, MAPK9, MSH6, TCF7 | 1.10E-06 |
|  | hsa-miR-221-3p | FOS, CASP3, CTNNB1 |  |
|  | hsa-miR-30a-5p | BAX, MLH1, JUN, CTNNB1, MAPK1 |  |
| p53 signaling pathway | hsa-miR-192-5p | ZMAT3, PERP, CDKN2A, MDM4, CCNE1 | 5.62E-05 |
|  | hsa-miR-221-3p | CASP3, BBC3, PTEN |  |
|  | hsa-miR-30a-5p | THBS1, BAX, TNFRSF10B, SERPINB5, RRM2 |  |
| Apoptosis | hsa-miR-192-5p | IL1RAP, IL1R1, CASP7, BCL2, PRKAR1A, IRAK1, IRAK2 | 2.47E-04 |
|  | hsa-miR-221-3p | TNFSF10, CASP3 |  |
|  | hsa-miR-30a-5p | CAPN2, BAX, PPP3CA, TNFRSF10B, BCL2L1 |  |
